# Replication of the Mammalian Genome by Replisomes Specific for Euchromatin and Heterochromatin

**DOI:** 10.3389/fcell.2021.729265

**Published:** 2021-08-31

**Authors:** Jing Zhang, Marina A. Bellani, Jing Huang, Ryan C. James, Durga Pokharel, Julia Gichimu, Himabindu Gali, Grant Stewart, Michael M. Seidman

**Affiliations:** ^1^Department of Neurosurgery, Institute for Advanced Study, Shanghai East Hospital, School of Medicine, Tongji University, Shanghai, China; ^2^Laboratory of Molecular Biology and Immunology, National Institute on Aging, National Institutes of Health, Baltimore, MD, United States; ^3^State Key Laboratory of Chemo/Biosensing and Chemometrics, College of Biology, Institute of Chemical Biology and Nanomedicine, Hunan University, Changsha, China; ^4^Department of Molecular Biology and Genetics, Cornell University, Ithaca, NY, United States; ^5^Horizon Discovery Group plc, Lafayette, CO, United States; ^6^Frederick National Laboratory for Cancer Research, Frederick, MD, United States; ^7^College of Medical and Dental Sciences, Institute of Cancer and Genomic Science, University of Birmingham, Birmingham, United Kingdom

**Keywords:** replication stress, replisome, CMG, FANCM, DONSON, GINS

## Abstract

Replisomes follow a schedule in which replication of DNA in euchromatin is early in S phase while sequences in heterochromatin replicate late. Impediments to DNA replication, referred to as replication stress, can stall replication forks triggering activation of the ATR kinase and downstream pathways. While there is substantial literature on the local consequences of replisome stalling–double strand breaks, reversed forks, or genomic rearrangements–there is limited understanding of the determinants of replisome stalling vs. continued progression. Although many proteins are recruited to stalled replisomes, current models assume a single species of “stressed” replisome, independent of genomic location. Here we describe our approach to visualizing replication fork encounters with the potent block imposed by a DNA interstrand crosslink (ICL) and our discovery of an unexpected pathway of replication restart (traverse) past an intact ICL. Additionally, we found two biochemically distinct replisomes distinguished by activity in different stages of S phase and chromatin environment. Each contains different proteins that contribute to ICL traverse.

## Introduction

**FIGURE 1:**
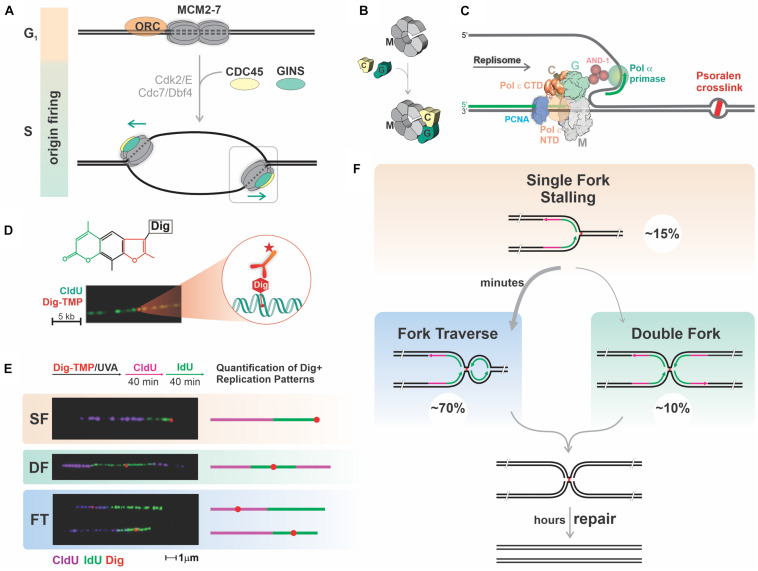
Mammalian replication forks faced with potent blocks restart DNA synthesis past the block, prioritizing replication over repair. **(A,B)** Several mechanisms ensure proper regulation of replication origin firing to prevent re-replication of chromosomal DNA. Origin licensing, the process of loading double hexamers of MCM2-7 (M) rings onto dsDNA at many potential origins, occurs exclusively in G1 phase under conditions that prevent initiation. Upon transition into S phase, some of these pre-replicative complexes are activated by association with CDC45 (C) and GINS (G) accompanied by melting of duplex DNA and locking of the MCM ring around the leading strand template. The temporal uncoupling of origin licensing and origin firing restricts replication to once per cell cycle. **(C)** DNA lesions covalently linking the two DNA strands pose a block to the CMG helicase pulling through the leading strand template. The replisome includes several proteins interacting with the CMG helicase, such as DNA Polymerases α, δ and ε (Pol ε Carboxy and N terminal domains are depicted), PCNA and CTF4. **(D)** Tagging of TMP with Digoxigenin (Dig-TMP) permits detection of a single ICL on a DNA fiber with Quantum-dot conjugated antibodies against the digoxigenin tag. **(E,F)** Quantification of replication patterns in the vicinity of ICLs: cells are treated with Dig-TMP/UVA and labeled with pulses of CldU and IdU, followed by DNA spreading, immunostaining, imaging, and quantification. Representative images and schemes of the replication patterns observed and their corresponding percentage. SF, single fork stalling; DF, converging double forks; FT, fork traverse. About 70% of the patterns correspond to fork traverse, which takes 5–6 min to complete. Fork traverse and double fork conversion would result in the same structure. Unhooking of the population of ICLs takes hours.

The replication machinery consists of a helicase to unwind parental strands and DNA polymerases and primase to synthesize daughter strands (Li et al., 2020). Replisomes also contain accessory factors that stabilize the association of the polymerases with DNA, contribute to the superstructure of the complex, and are important for initiation of replication (Bai et al., 2017; Douglas et al., 2018; Baretić et al., 2020; Li et al., 2020). The helicase contains a six subunit off-set open ring structure formed by the MCM (M) proteins and is loaded on duplex DNA, only in G1 phase, at sites that may become origins of replication (origin licensing) (Deegan and Diffley, 2016). In S phase MCM complexes accumulate additional proteins, including CDC45 (C) and the four GINS (G) proteins. This association is accompanied by localized DNA melting, locking of the MCM ring around the template strand for leading strand synthesis, and activation of the CMG helicase (origin firing). While the locked ring confers resistance to detachment it would seem to pose insurmountable problems when the replisome encounters large impediments (Figures 1A,B).

Replication stress is imposed by blocking either the CMG or the DNA polymerases. Most experiments target the polymerases taking advantage of drugs that are direct inhibitors or suppress nucleotide triphosphate synthesis (Schwab et al., 2010; Whalen and Freudenreich, 2020). However, this strategy cannot report the consequences of replisome encounters with helicase blocks. To model these events, we developed an experimental approach based on interstrand crosslinks (ICLs), always considered impassable blocks to replication (Marmur and Grossman, 1961) and potent inducers of replication stress (Vesela et al., 2017; Renaudin and Rosselli, 2020). Crosslinking agents are highly toxic to growing cells and are frequently used in cancer chemotherapy (Rycenga and Long, 2018).

Understanding replication dependent ICL removal in mammalian cells was a considerable challenge for decades. Most models described stalling of a replisome at an ICL followed by unlinking of the duplex strands (unhooking) after which the replication fork could be rebuilt to allow resumption of synthesis (Kuraoka et al., 2000; Muniandy et al., 2010). Although genes were identified as being important for repair, notably those linked to Fanconi Anemia, there was little insight regarding events following fork encounters with ICLs. This changed with the development by the Walter group of a Xenopus egg extract system which supported replication of a plasmid with a site-specific crosslink. They observed that replication was completed on either side of the ICL before unhooking (Raschle et al., 2008) and that repair occurred after replication on both sides of the ICL was concluded (Zhang et al., 2015). Their observations have been very influential and their model has replaced earlier depictions of ICL repair.

Although the Xenopus extract system is very powerful, the extent to which it recapitulates replication fork encounters with genomic ICLs in living mammalian cells is unclear. To address this, we designed a strategy based on DNA fiber technology (Schwab and Niedzwiedz, 2011). Although this technology has been applied to studies investigating the influence of DNA damaging agents on DNA replication (Merrick et al., 2004; Elvers et al., 2011; Li et al., 2018), it was not possible to distinguish between a global response to stress vs. local effects due to fork encounters with a DNA adduct. To overcome this limitation we exploited the properties of psoralens, which are photoactive crosslinking compounds (Hearst et al., 1984). Psoralens form a high frequency of ICLs, more than 90% with the trimethyl psoralen (TMP) used in our experiments (Lai et al., 2008; Muniandy et al., 2010), and can be conjugated to an antigen tag without altering the crosslink: monoadduct ratio (Huang et al., 2013).

## Results and Discussion

### Replication Tract Encounters With Digoxigenin Tagged Trimethyl Psoralen

To visualize ICLs we linked TMP to digoxigenin, frequently used as an immunotag (Figures 1C,D; Thazhathveetil et al., 2007). Cells were incubated with Digoxigenin Tagged Trimethyl Psoralen (Dig-TMP), exposed to long wave UV (UVA), and pulsed successively with nucleoside analogs to label newly synthesized DNA. Replication tracts were displayed on DNA fibers by immunofluorescence against the analogs. The ICLs were visualized by immunoquantum dot detection (Simons et al., 2015; Kong et al., 2016). Less than 10% of tracts had an encounter, and, as anticipated, we observed both single and double fork stalling events at ICLs (Raschle et al., 2008). Notably, however, a major outcome of our analysis, one that we termed replication traverse, was the restart of DNA synthesis past intact ICLs (Figures 1E,F; Huang et al., 2013). While replication restart past monoadduct blocks has been known for many years (Rupp and Howard-Flanders, 1968; Heller and Marians, 2006; Lehmann and Fuchs, 2006; Taylor and Yeeles, 2018; Guilliam and Yeeles, 2020), our observations were contrary to over 50 years of conventional wisdom (Marmur and Grossman, 1961). However, ICL traverse has been confirmed by recent work from other laboratories (Mutreja et al., 2018; González-Acosta et al., 2021).

Comparison of the lengths of tracts with or without ICL encounters indicated that traverse required only a few minutes. We also found that ICLs embedded in replication tracts were unhooked (first repair step) over a period of several hours. Although the time required for unhooking an individual ICL is not known, it is apparent that resolving the population of replication associated ICLs occurs over a much longer time than traverse (Huang et al., 2019).

The Walter group showed that the immediate product of double fork collisions on either side of an ICL was an “X” structure. This is also the product of ICL traverse once Okazaki fragment ligation has occurred (Huang et al., 2013; Zhang and Walter, 2014; Figure 1F). Consequently, the traverse pathway and the less frequent double fork collisions provide options for completing replication on the distal side of a block. Relative to a stalled single fork, the much greater frequency of these two options points to an evolutionary cost benefit analysis that favors the completion of S phase over removal of the impediment. We have proposed the term “replication imperative” to characterize the priority of replication over lesion repair (Yang et al., 2019).

### ATR and FANCM Are Important for Replication Traverse of ICLs

Replication stress activates the damage responsive kinase, ataxia telangiectasia and Rad3-related (ATR), which has hundreds of substrates, including MCM proteins and those involved in restarting stalled forks (Cortez et al., 2004; Matsuoka et al., 2007). The embryonic lethality of ATR knockout mice (O’Driscoll, 2009) emphasizes the importance of the response pathways to cell and organismal viability. Inhibition of ATR completely suppressed ICL traverse indicating that it was a component of the ATR response to replication stress.

The traverse pathway was partially dependent on the activity of the DNA translocase FANCM, a substrate of ATR (Huang et al., 2013). Expression of a phospho-resistant, or a translocase inactive, form of FANCM in a FANCM knockout cell, reduced traverse frequencies to levels equivalent to those displayed by the knockout cells (Huang et al., 2019). Thus, the traverse option was dependent on a translocase activity under ATR control. It should be noted that the CMG helicase has no translocase activity while FANCM has no helicase activity (Meetei et al., 2005).

Chromatin Immunoprecipitation (ChIP) against FANCM from cells exposed to TMP/UVA demonstrated an interaction with replisome proteins. Also recovered was MCM2 phosphorylated at Serine 108, a site of ATR-dependent phosphorylation and a marker of a “stressed” replisome (Cortez et al., 2004). Importantly, incubation of cells with an ATR inhibitor eliminated pMCM2S108 and abolished the interaction between FANCM and the replisome (Huang et al., 2019).

### Loss of the GINS in ICL Proximal Replisomes

The locked ring structure of the active replisome and the prohibition on replisome loading during S phase raised questions about replisome composition following collisions with ICLs. We identified a replisome complex in TMP/UVA treated cells containing FANCM, pMCM2S108, but not the GINS. Notably, the loss of the GINS complex was not affected by translocase defective FANCM. Thus, it was possible to split the role of FANCM into two stages: the displacement of the GINS requiring ATR dependent association with the stressed replisome; the restart of replication, dependent on the translocase function (Huang et al., 2019).

Proximity Ligation Assays (PLA) (Koos et al., 2014) reported the interaction of MCM2 or pMCM2 and the Dig tag on the ICLs, while the PLA between the GINS proteins and the tag remained at background levels. Furthermore, as expected, in cells treated with an ATR inhibitor there was an increase in PLA signal between MCM2 and the ICL and a greatly increased frequency of GINS proximal to ICLs.

**FIGURE 2:**
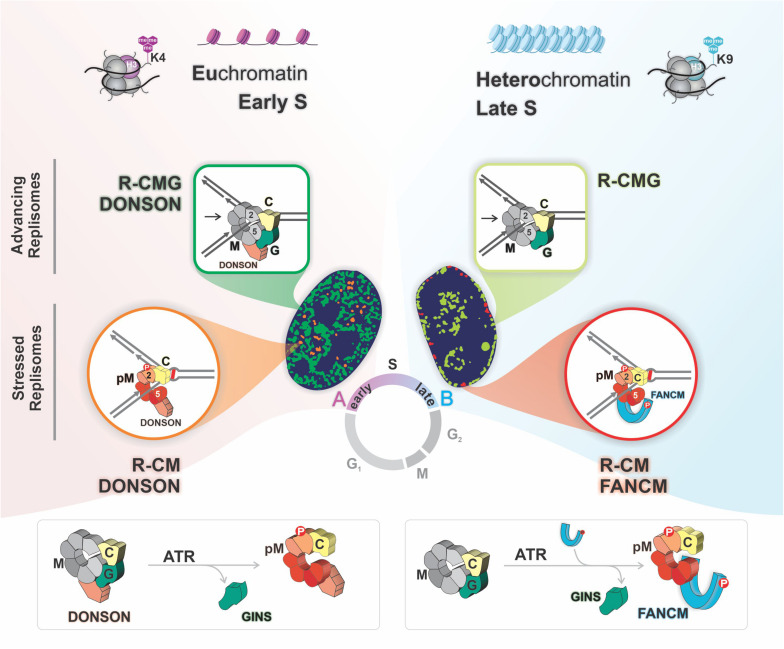
Replisomes with alternative compositions are active in the two chromatin compartments. There are two advancing replisomes, one with DONSON (R-CMG DONSON), biased toward early replicating euchromatin, and one without (R-CMG), preferentially localized to late replicating heterochromatin. In euchromatin the encounter with an ICL triggers MCM2 phosphorylation on serine 108 by ATR, and eviction of the GINS proteins yielding R-CM DONSON. In heterochromatin there is an ATR dependent recruitment of FANCM which is required for the loss of the GINS and the formation of R-CM FANCM.

These results demonstrated that ICL proximal replisomes, marked by pMCM2, lacked the GINS complex. In addition, the increased proximity of GINS containing replisomes to ICLs following the inhibition ATR is indicative of the accumulation of GINS associated replisomes stalled at ICLs, implying the loss of an ATR-dependent mechanism to release the structural constraints of the CMG. These observations were consistent with a model in which, upon encounters with ICLs, replisomes lose the GINS complex, thus unlocking the CMG ring during the few minutes required for traverse. In the absence of ATR, FANCM is not recruited, there is no traverse, and the GINS complex is retained on replisomes that accumulate at the ICLs (Figure 2, lower right panel).

### DONSON Contributes to Replication Traverse of ICLs

Our finding that while traverse events were entirely dependent on ATR but only approximately 50% of these depended on FANCM suggested that cells contained another pathway to restart replication. After testing of several candidate proteins we found that traverse frequencies were reduced in cells deficient in DONSON (downstream neighbor of Son) protein, a constitutive replisome component (Evrony et al., 2017; Reynolds et al., 2017). Double knockdown of both DONSON and FANCM revealed a decline greater in ICL traverse than with either individual deficiency, indicating that they functioned in separate pathways. PLA analysis indicated that DONSON was proximal to ICLs and the signal frequency rose upon ATR inhibition. These results were consistent with DONSON being retained on replisomes transiently proximal to ICLs, unlike the GINS proteins. Furthermore, following ATR inhibition there was an accumulation of DONSON containing replisomes stalled at ICLs (Zhang et al., 2020).

The presence of replisomes containing DONSON and/or FANCM raised the question of whether they resided within the same or separate replisome complexes. To address this, we prepared chromatin from cells exposed to TMP/UVA. After digestion of the DNA, the solution was cleared of “non-stressed” replisomes by immunoprecipitation against a GINS protein. Then DONSON bound complexes were recovered from the supernatant, after which FANCM associated complexes were captured from the residual supernatant. DONSON was present in both GINS positive and negative replisomes, the latter marked as stressed replisomes by pMCM2S108. FANCM coprecipitated with replisomes that also contained pMCM2S108, but not DONSON. These results were confirmed by PLA. Thus, there were separate and distinguishable stressed replisomes containing either DONSON or FANCM but not both. Furthermore, DONSON clearly had a different role than FANCM because it was associated with both stressed and unstressed replisomes while FANCM was associated only with stressed replisomes (Zhang et al., 2020). This argued against the assumption of a single species of stressed replisome and raised the question: Do these different replisome complexes exist in the same cell at the same time?

To answer this, we performed a sequential PLA experiment in cells exposed to TMP/UVA. After PLA between DONSON and pMCM2S108 the cells were imaged, stripped, and PLA between FANCM and pMCM2S108 performed. Alignment of the first and second images of the same cell demonstrated that the complexes could reside within the same cell at the same time but not at the same place. The frequency of DONSON: pMCM2S108 was biased toward early S phase while FANCM: pMCM2S108 strongly favored late S phase.

Analysis of the DNA sequences associated with the two stressed replisomes supported this conclusion. Alu sequences replicate in early S phase and were found in the DONSON fraction, while Satellite 3 sequences replicate late and were captured in the FANCM fraction. ChIP and PLA analyses of DONSON: H3K4me3 (a euchromatin marker) and FANCM: H3K9me3 (heterochromatin marker) confirmed the localization of the DONSON stressed replisome to predominantly euchromatin while the FANCM-containing stressed replisome was more frequently localized within heterochromatin.

DONSON was originally described as a replisome component in unstressed cells (Reynolds et al., 2017). Consequently, it was of interest to ask about the distribution of DONSON replisomes in cells without treatment with a DNA reactive agent. We again found the same bias toward euchromatin and early S phase as above. FANCM associated replisomes were heterochromatic and were more active in late S phase. The FANCM signal frequency was much lower than in cells with ICL induced replication stress and was likely due to “spontaneous” replisome impediments. DNA fragments bound by the DONSON complex were preferentially located in early replicating regions and in euchromatin, while the FANCM associated sequences were strongly biased toward late replicating regions and heterochromatin.

### Outstanding Questions

#### Why Two Replisomes?

We suggest that the answer lies in the differences between eu- and heterochromatin. Replisomes in euchromatin are more likely to encounter DNA damage (Takata et al., 2013), transcription complexes, and R loops (Hamperl et al., 2017). Deficiencies in DONSON would be expected to adversely influence the response to replication stress in these areas of the genome. DONSON is a member of a group of replication associated proteins, mutations in which result in microcephaly and dwarfism (Bicknell et al., 2011; Evrony et al., 2017; Reynolds et al., 2017; Van Esch et al., 2019; Cicconi et al., 2020; Matos-Rodrigues et al., 2020; Starokadomskyy et al., 2021). Compromised replication through genomic areas with active transcription could have a negative impact on completing S phase and consequently, cell number, resulting in smaller brain and body size. Additional pathology may be derived from stalled replication forks that can activate inflammatory responses through the elaboration of DNA fragments that enter the cytoplasm and stimulate interferon pathways (Ardeljan et al., 2020).

In contrast to DONSON, FANCM does not appear to be a constitutive replisome component. Instead, it is preferentially recruited to replisomes stalled in heterochromatin, most likely at “difficult to replicate” sequences during late S phase (Janssen et al., 2018). FANCM has homologs in archaea (Meetei et al., 2005), and may have evolved, in part, to assist replisomes duplicating sequences with an inclination to block replication. In disorders with mutant FANCM (Bogliolo et al., 2018; Catucci et al., 2018) we would predict an exacerbation of replication stress in regions of heterochromatin (Nikolov and Taddei, 2016).

#### What Is the Mechanism of Traverse?

Our proposal of restart of replication past ICLs is based on an interpretation of the pattern of nucleoside analog incorporation in DNA fibers. However, these patterns cannot distinguish between multiple explanations for the incorporation. The identification of the molecular machinery responsible for replication traverse of the ICLs is a key question awaiting answer. Some relevant considerations are:

(1) Parental strand replacement synthesis. Standard fiber patterns cannot distinguish between synthesis of daughter DNA strands or replacement synthesis of a parental strand (“nick translation” of the strand). However, in experiments in which parental strands were differentially marked, we have not observed any replacement synthesis (Huang et al., 2013, 2019).
(2) Extension synthesis primed by RNA in an R loop has been described in *Escherichia coli* (Camps and Loeb, 2005). Treatment of cells with RNA polymerase inhibitors blocks R loop formation (García-Muse and Aguilera, 2019) but had no effect on traverse frequencies. Furthermore, deficiencies in FANCM increase the frequency of R loops (Schwab et al., 2015), but we found that traverse frequencies declined in FANCM mutant or knockout cells.
(3) Is the restart synthesis due to a CMG replisome? Replication traverse of ICLs is inconsistent with an irreversibly locked CMG. We do not know if a CMG that encounters an ICL drives DNA synthesis on the distal side. If so, a gate must transiently open and close. Recent work implies reversible gates in replisomes (Yardimci et al., 2012; Gao et al., 2019) suggesting a mechanism to permit passage across large impediments. There may be more than one gate as the MCM2-MCM5 gate (closed by GINS and CDC45 and used for origin licensing) was not opened in the recent analysis of the CMG gate involved in transitions between single and double strand DNA binding (Wasserman et al., 2019). Furthermore, the GINS were not lost in the Walter group’s characterization of replisome movement past a bulky protein adduct (Sparks et al., 2019). The relationship between these results and events in a live cell in which a stalled replisome activates an ATR cascade remains to be determined.
(4) Restart of replication would require priming downstream of the ICL. Recently the Mendez lab described the requirement of the PrimPol primase for about 50% of traverse events (González-Acosta et al., 2021). While these results identify PrimPol as important for traverse they also argue that there are other factors that support repriming downstream of an ICL.
(5) The Lopes group has suggested a requirement for replication fork reversal prior to ICL traverse (Mutreja et al., 2018). Reversal of a replication fork after an encounter would restore duplex DNA to the proximal as well as distal side of an ICL (Kondratick et al., 2021). One of the rationales for fork reversal is that it allows for resolution of the impediment. However, the ICLs were intact at the time of traverse. Consequently, while ICLs might provoke fork reversal it is not clear what contribution this would make to the restart process. One way to assess the relevance of fork reversal to traverse would be to perform the fiber assay in cells deficient in key reversal factors such as RAD51, ZRANB3, and SMARCAL1. These experiments are underway.

Finally, we note the difficulty of addressing many of the mechanistic questions raised by the traverse phenomenon. While the powerful system developed by the Walter group would seem ideal for this inquiry, the restart pathway does not occur in Xenopus egg extracts. Early stage replication in frog embryos is very rapid as a result of many origins with short distances between them. This would favor double fork convergence at ICLs and there may be no need for the traverse option (Semlow and Walter, 2021). Elucidation of the effectors of the molecular steps of traverse will require an assay system that can distinguish fork proximal and distal sides of an ICL. The resolution of current fiber assays is far from adequate and new assays will need to be developed to satisfactorily address these questions.

## Data Availability Statement

The original contributions presented in the study are included in the article/supplementary material, further inquiries can be directed to the corresponding author/s.

## Author Contributions

JZ, MAB, RCJ, JH, DP, JG, and HG performed the experiments and generated the concepts derived from their experimental work. MAB prepared the figures. MAB, GS, and MMS wrote and edited the manuscript. All authors contributed to the article and approved the submitted version.

## Conflict of Interest

DP was employed by company Horizon Discovery Group plc. The remaining authors declare that the research was conducted in the absence of any commercial or financial relationships that could be construed as a potential conflict of interest.

## Publisher’s Note

All claims expressed in this article are solely those of the authors and do not necessarily represent those of their affiliated organizations, or those of the publisher, the editors and the reviewers. Any product that may be evaluated in this article, or claim that may be made by its manufacturer, is not guaranteed or endorsed by the publisher.
